# Items and Remarks

**Published:** 1873-10

**Authors:** E. N. Brush


### Items and Remarks.

BY E. N. BRUSH.

The popular Summer resort, "Desert Island," off the coast of Maine, has been visited by an epidemic; several persons have been attacked, a few with fatal result. The cause is assigned to impure water and surface drainage.——The subject of enlarging the capacity of our reservoir is under consideration. If the present one is not large enough to supply the wants of the city, why not supply the eastern and southern portions by pumping direct from the Hamburgh Canal, as the quality of the water does not seem to be improved much by passing through the reservoir. We can tell when the tanks of a certain oil refinery, situated at the lower end of the canal have burst by the taste of our *Niagara* (?) water, and not believing in high dilutions, we prefer to take our oil in the pure state if we must take it at all.——In a communication to the Academy of Medicine, Paris, on some observations on Septicæneia in Man, M Davaine says: Recently the study of the conditions in which certain epidemics of typhoid fever have been produced, throws new light upon the relations of this disease with the miasms or emanations of putrefied organic substances. Numerous facts, observed in France, Switzerland, Germany, and especially in England and America, have abundantly demonstrated these relations. Numerous epidemics of typhoid fever have been determined by infiltrations from water closets or sewers into drinking-water.——Dr. Ollier, of Orleans, reports (*Revue Med. Francaise et Etrangere*) a case of Ovariotomy, followed by twice pregnancy. The woman became pregnant three months after the operation and was delivered at full term.——Dr. Dickinson has been recently experimenting with Phosphorus in cases of nervous affection, in which there was deficiency of nervous energy, and has obtained decided evidence of its value. He administers it dissolved in oil or lard, enclosed in gelatine capsules, the dose being 1-30 of a grain two or three times daily, after food always.—*Practitioner*.——Of four hundred and forty-three candidates for membership, of the Royal College of Surgeons, of England, one hundred and four were rejected.——Dr. Mynert reports in the *Lyon Medicale* the death of a patient at eighty-five, who had been confined four times, the first time at the age of forty, the second at forty-eight, the third at fifty-one and the fourth at fifty-six.—*Med. Times.*——A call has been issued for a meeting of the Surgeons of the late Confederate Army, at Atlanta, Ga., May 20th, 1874. It is the object of this meeting to collect and preserve in some suitable form the medical and surgical observations made in the Confederate service, which would otherwise be lost to science.——Dr. E. S. O'Grady, of Mercer's Hospital, England, recently ligated the innominate artery for an anuerism of the subclavian in a patient nearly sixty years of age. —*Record.* We have seen no report of the final result.——Prof. W. A. Hammond has resigned his Professorship in Bellevue Hospital Medical College Dr. Edward Janeway will lecture on Materia Medica and Therapeutics. The lecture on diseases of the nervous system will be given by the Professor of Principles and Practice of Medicine.——In view of the approaching era of political speech-making, a lover of his race, advertises for some secret and expeditious method of communicating lock-jaw.—*Record.*——The Canada *Lancet* comes to us much enlarged and changed in form, having adopted the style of the *Medical Record*, *Times*, and other semi-monthly and weekly journals. It is still issued monthly, but the editor hopes soon to present a semi-monthly or weekly edition.——Dr. Bowling, of the *Nashville Journal*, replies to Dr. Davis, of the *Chicago Medical Examiner*, regarding the proper diet during an epidemic of cholera in a manner which will afford some good after dinner reading for those who enjoy a hearty laugh. His definition of animal products is amusing in the extreme. The article ends with bill of fare, which seems sufficient for any period, whether an epidemic of cholera is present or not.——Specialists seem to be appreciated in England if we are to judge from the following which we take from the lecture of Dr. Robert Barnes on the Convulsive Diseases of Women, reported in the *Lancet.* He says. I have recently been honored by a visit from a lady of typical modern intelligence, who consulted about a fibroid tumor of the uterus; and lest I should stray beyond my business, she was careful to tell me that Dr. Brown-Sequard had charge of her nervous system; that her abdominal organs were entrusted to Sir William Gull; that Mr. Spencer Wells looked after her rectum, and that Dr. Walshe had her heart.——Following a "pathy" often leads a man to do strange things in medicine, and none, we believe, are more ridiculous than those performed by some of the believers in, and followers of Hahnemann. A young man of our acquaintance was recently asked by a friend to see her mother, who was "bleeding to death." On entering the room he found the patient suffering from severe hemorrhage from a ruptured vein in a varicose ulcer. Pressure had been moderately applied by some one above the ulcer and compress and bandage over it. On removing the bandage, six or eight Homeopathic pills fell out which had been placed between it and the compress by a Homeopathic physician, who, he then learned, had seen the case, and attempted to control the hemorrhage. Slight pressure, properly applied, soon arrested all bleeding, and our friend went his way meditating on the capability which some minds possess of embracing the most absurd beliefs.
——We copy the following from an Eastern paper. "James Foot, Esq., of this town, lost a valuable cow last week by congestion of the lungs, and, having another taken in the same way, immediately summoned Dr. Herman D. Burghardt, who administered Homeopathic remedies with such skill that the cow is convalescent, and bids fair to be in the 'cream business' again soon." Will the cream be effected in any way by the remedies administered, and if so will it not be dangerous for any one to use it, as it will, of course, be a "high dilution," and consequently very strong.——We acknowledge the receipt of two new Medical Journals (Eclectic) *The Medical Review*, of Indianapolis, and *The Medical Eclectic*, of New York. The editors of the *Review* bewail the fact that while political factions can clasp hands "across the bloody chasm," reconciliation is not for doctors, and are of the opinion that the recent resolutions introduced by certain members of the Oneida County Medical Society, advocating consultation with irregular practitioners, caused the Editor of this JOURNAL to "spontaneously and incontinently explode." Allow us to correct a misapprehension. The Oneida County Society did not "*focus*" on the aforesaid resolutions, but voted them down almost unanimously. The article by Dr. Derby, entitled "What Stretched," does not reflect much credit upon Dr. Milton Jay, of Chicago, whose report of two inches elongation in a fractured femur was noticed in the August numbe of this JOURNAL.——In a report of a recent meeting of the Dublin Obstetrical Society in the *Obstetrical Journal of GreatBritain andIreland*, we notice a discussion concerning inversion of the Uterus. Several successful cases are reported and some very interesting remarks made. We are surprised, however, at the opinion expressed by the President of the society, Dr. Evory Kennedy, as follows: "He was not aware of reduction having been effected after fifteen years. He thought these were very rare cases. He thought the condition of the tissues would so alter in the course of ten or fifteen years that it would be totally impossible to effect reduction at the end of that time. He could lay his hand at that moment on more than one case of inversion of the uterus, where the constitution had become used to it, and where the period of life had arrived when it ceased to be any inconvenience to the individual. He had in his room within the last six weeks a patient whom he first saw some twenty-six years ago She came to him with inversion of the Uterus. She was exceedingly bloodless and anæmic. It was before the days of chloroform. He made repeated efforts by pressure upwards and backwards, and by squeezing to reduce the Uterus, but failed. * * * * * * * She was now a fine healthy woman, sixty years of age, and had no trace of disease about her. She had become reconciled to it, and the Uterus had to a great extent become absorbed." We are not surprised that the Doctor should have failed in this case to affect reduction; failure will occasionally be the lot of all; but, that he should express such lack of faith in the possibility of reduction after ten or fifteen years, after the achievements in his own country by Dr. Tyler Smith, and the still larger experience in this country of Prof. James P. White. In the eleven cases which have fallen under Prof. White's care, every one of which has been reduced, the time which had elapsed since inversion, has varied from a few moments to twenty-two years, showing that in all its stages reduction of the inverted Uterus is possible, and should, in all instances, be attempted. The Doctor's attention has probably never been called to the cases reported by Dr. White.

